# “For normal people, it just doesn’t work”: uncovering popular lenses on environmental sustainability in The Netherlands

**DOI:** 10.1080/15487733.2025.2500153

**Published:** 2025-05-13

**Authors:** Kjell Noordzij, Joost Oude Groeniger, Willem de Koster, Jeroen van der Waal

**Affiliations:** ^a^ Department of Public Administration and Sociology, Erasmus University Rotterdam, Rotterdam, the Netherlands; ^b^ Department of Public Health, Erasmus MC, Rotterdam, the Netherlands

**Keywords:** Sustainability, environmental attitudes, climate change, education, focus-group interviews

## Abstract

Governments grapple with garnering public support for the interventions they propose to achieve with respect to environmental sustainability. We depart from the position that interventions for these issues must align with public perspectives to receive support among the population at large. Our study uses 14 focus-group interviews (*n* = 57) conducted in the Netherlands to inductively explore these perspectives on a wide range of sustainability issues and their interventions. Our sample predominantly comprises individuals who have not completed tertiary education. As extant research suggests, these individuals are less concerned with sustainability issues. We identify three lenses through which our respondents perceived sustainability, namely how it: 1) impacts their personal life (*lens of need*); 2) aligns with their community (*lens of community*); and 3) raises doubts (*lens of doubt*). They reveal that many respondents are principally engaged with sustainability in ways that they felt had practical repercussions for them or their community. We conclude by instigating a rethinking of inclusive sustainability interventions by sensitizing them to the perspectives individuals have of sustainability issues and interventions, and those of non-tertiary educated individuals in particular.

## Introduction

Environmental sustainability has become central in public and political debate in many contemporary Western societies. Sustainability concerns issues of the physical environment (such as forests) and climate (such as global warming) and related challenges (such as chemical pollution or carbon-dioxide (CO_2_) emissions), as well as the interventions designed to address them (such as waste-separation systems or solar panels) (cf. Dunlap 2010). Because the legitimacy and effectiveness of these interventions are shaped by public support, it is crucial to map the diversity in how individuals engage with sustainability issues and interventions.

Research has identified numerous ways in which different social groups interact with sustainability, with the engagement of individuals without tertiary education standing out. Survey studies show that this demographic is, for example, typically less concerned about climate change and its risks (see, e.g., Hoekstra et al. 2024; Hornsey et al. 2016; Kvaløy, Finseraas, and Listhaug 2012; Lee et al. 2015; Lübke 2022), is less supportive of renewable energy interventions (see, e.g., Liebe and Dobers 2019), and is less focused on other sustainability issues as well, such as environmental protection (see, e.g., Kennedy and Givens 2019). Turning to their behavior, on the one hand, studies have found that this sociodemographic group tends to engage less in, for example, sustainable transport or sustainable shopping (see, e.g., Geerts, Vandermoere, and Oosterlynck 2023; Quaglione et al. 2019) and to be less aware of mitigation strategies to deal with climate-change related events (see, e.g., Pisello et al. 2017). On the other hand, research has demonstrated that individuals in a weaker economic position, which many non-tertiary educated individuals are, display sustainable behavior out of economic necessity, like recycling (see, e.g., Laidley 2013).

Especially considering that the conclusions of extant studies are predominantly based on survey research, a more in-depth study is warranted on how non-tertiary educated individuals *themselves* perceive sustainability issues and interventions. Our study, therefore, aims to better understand how this majority demographic—accounting for about 63% of the Dutch population, according to Statistics Netherlands (2024a)—engages with environmental sustainability. In other words, we aim to inductively uncover *popular perspectives* on sustainability. To do so, we predominantly sampled individuals who had not undertaken tertiary education and conducted in-depth interviews with 57 individuals in 14 focus groups.

Sustainability has been part of public and political debate in the Netherlands for decades (Martens and Spaargaren 2005) and has more recently become a politically polarized issue among the public at large (see, e.g., Rekker and Harteveld 2024). Additionally, different levels of government are devoting substantial shares of their resources to implementing sustainability interventions that individuals are asked to support, adopt, or participate in (see, e.g., Ministerie van Economische Zaken en Klimaat 2023; VNG 2023; see, also, Ebrahimigharehbaghi et al. 2019; Jansma, Gosselt, and de Jong 2020). This context makes it likely that many people will have crystallized perspectives on sustainability and have encountered governmental attempts to implement interventions. We asked respondents to reflect on a wide range of sustainability issues (e.g., renewable energy, green environments, pollution) and potential interventions (e.g., wind turbines, gardens, recycling). The interviews were conducted in six municipalities, both rural and urban, and sustainability interventions introduced in these municipalities were used as elicitation material to encourage respondents to think about more inclusive interventions.

We connect our findings to a diverse body of studies in environmental sociology, which analyze “societal-environmental relations or interactions” and “the continued struggles and debates over how to deal with [them]” (Dunlap 2010, 15–16). Our approach adds to this literature by studying individuals’ perspectives through their own eyes while accounting for the multifaceted nature of these perspectives across a wide range of sustainability issues and interventions.

The first of these contributions enables us to produce an in-depth interpretation of how individuals perceive sustainability—and how different individuals might hold different perspectives. Consequently, our focus “is no longer [on] *preferences* but *perspectives*,” which “are the lenses through which people view issues” (Cramer Walsh 2004, 2; italics in original). By putting these lenses center stage, we unearth a rich and encompassing understanding of sustainability “on the ground and in the lifeworld, recognizing the multiple, uneven layers of…meaning and experience” (Baker 2019, 98). By developing an in-depth understanding of how individuals *themselves* perceive sustainability issues and interventions, and by anticipating that these perspectives are diverse and multifaceted (see, e.g., Jansma, Gosselt, and de Jong 2020), we locate these perspectives in both the context of their social and cultural life-worlds as well as their material conditions and physical surroundings, which are often analyzed separately in environmental sociology (Dunlap 2010). Moreover, by speaking to individuals themselves about sustainability, we uncover a much more in-depth understanding of *why* they support sustainability issues and interventions, and under what conditions.

Our second contribution is that we explore individuals’ multifaceted perspectives on sustainability while also being sensitive to how these differ or compare across a wide range of issues and interventions. This advances on extant literature that commonly studies levels, determinants, or the nature of support for these issues and interventions in isolation, likely because “‘the environment’ is an enormously complex phenomenon, open to highly diverse conceptualizations and operationalizations [which] is a key factor in generating diversity among environmental sociologists” (Dunlap 2010, 16). Studies to date have explored issues like climate change (see, e.g., Hoekstra et al. 2024; Hornsey et al. 2016), energy-efficiency renovations (see, e.g., Ebrahimigharehbaghi et al. 2019; Jansma, Gosselt, and de Jong 2020), renewable energy generation (see, e.g., Corner et al. 2011; Liebe and Dobers 2019), consumption (see, e.g., Carfagna et al. 2014; Hinton and Goodman 2010), and mobility (see, e.g., Quaglione et al. 2019), but authors only sparsely analyze support for sustainability across a wider range of issues (cf. Fritz et al. 2021; Kennedy and Givens 2019). Studying sustainability issues and interventions in isolation, however, complicates insight into how far individuals’ perspectives travel across different issues and interventions or whether unearthed perspectives are specific to the issue or intervention at hand. Our approach, instead, is well-versed in providing such insight.

Below, we describe our method and data in more detail. Afterward, we present our findings, discussing the three popular perspectives we inductively uncovered. We conclude by discussing the theoretical and practical implications of these results, highlighting relevant ventures for future research and the implications of our findings for professionals and governments.

## Method and data

We conducted 14 focus-group interviews during the period January to May 2023 involving 57 individuals. The vast majority of our respondents had not completed a tertiary education (i.e., a university or a university of applied sciences): only five had finished their education at the university of applied sciences level; of the further five who were still studying, only one was doing so at a university.^1^ Most of the respondents were either retired, unemployed, or had working-class occupations.

We used multiple sampling strategies to recruit potential respondents. The most successful were door-to-door flyering, advertising in local news outlets, and contacting local associations and clubs. Each strategy presented brief information on the study’s goal, the interview procedure, and the compensation awarded for participation. We emphasized our interest in recruiting respondents who had not completed a tertiary education. Our focus was on six specific municipalities, and so we informed potential respondents that they needed to live in one of these municipalities.^2^ A minimum of two group interviews in each municipality proved sufficient for achieving theoretical saturation.

Potential respondents were selected based on their level of education and municipality. We ensured that a balance was obtained with respect to age, gender, and ethnicity by sampling in relation to specific demographics (e.g., through youth associations). Table 1 provides details on the final sample. We went to great lengths to ensure that the respondents felt comfortable and free to express their views. We asked the sampled respondents to select the setting, date, and location of the interview. They were also asked to invite other potential respondents to attend so that the interviews would resemble “informal talk” (Cramer Walsh 2004, 1) and the respondents would feel comfortable giving their opinions in the presence of an interviewer (see, also, Noordzij, de Koster, and van der Waal 2021). As the respondents were the ones in charge of inviting other respondents, a small number of focus groups contained only two individuals. All the respondents provided their written informed consent, with the form giving them information about our study and their rights as respondents.

**Table 1.** Overview of the interviews and the background characteristics of the respondents (*n* = 57).

Pseudonym	Gender	Age	Municipality	Current occupation^a^	Migration background^b^
*Interview 1 (location: welfare organization; approx. 40 min)*					
Maria	Female	63	Rotterdam	Unemployed	No
Niloofar	Female	45	Rotterdam	Unemployed	Yes
Aya	Female	52	Rotterdam	Unemployed	Yes
Firuza	Female	57	Rotterdam	Unemployed	Yes
Yasamin	Female	23	Rotterdam	Unemployed	Yes
*Interview 2 (location: community center; approx. 60 min)*					
Trijntje	Female	74	Lansingerland	Pensioner	No
Sandra	Female	55	Lansingerland	Unemployed	No
Aaltje	Female	77	Lansingerland	Pensioner	No
Paulina	Female	78	Lansingerland	Pensioner	No
Anna	Female	70	Lansingerland	Pensioner	No
*Interview 3 (location: community center; approx. 70 min)*					
Monica	Female	52	The Hague	Unemployed	No
Lia	Female	61	The Hague	Unemployed	Yes
Anita	Female	58	The Hague	Unemployed	No
*Interview 4 (location: living room; approx. 60 min)*					
Yvonne	Female	67	Voorne aan Zee	Pensioner	No
Simone^c^	Female	55	Voorne aan Zee	Youth worker	No
Antoinette	Female	62	Voorne aan Zee	Customs officer	No
Edith	Female	64	Voorne aan Zee	Wedding official	No
*Interview 5 (location: community center; approx. 70 min)*					
Bernard	Male	80	Lansingerland	Pensioner	No
Frank	Male	76	Lansingerland	Pensioner	No
Peter	Male	65	Lansingerland	Pensioner	No
Robert	Male	78	Lansingerland	Pensioner	No
*Interview 6 (location: community center; approx. 40 min)*					
Martha	Female	60	Voorne aan Zee	Unemployed	No
Jacqueline	Female	56	Voorne aan Zee	Unemployed	No
Irma	Female	68	Voorne aan Zee	Pensioner	No
Albert	Male	66	Voorne aan Zee	Pensioner	No
Dirk	Male	78	Rotterdam	Pensioner	No
*Interview 7 (location: canteen; approx. 40 min)*					
Marcel	Male	53	The Hague	Public area cleaner	No
Jeffrey^c^	Male	31	The Hague	Team leader public area cleaner	No
Selam	Male	37	The Hague	Public area cleaner	Yes
Ronald	Male	49	The Hague	Public area cleaner	No
*Interview 8 (location: cultural center; approx. 70 min)*					
Hendrina	Female	71	Voorne aan Zee	Pensioner	No
Ingrid^c^	Female	60	Voorne aan Zee	Unemployed	No
Marja^c^	Female	66	Voorne aan Zee	Pensioner	No
Inge	Female	55	Voorne aan Zee	Mental health worker	No
*Interview 9 (location: garden shed; approx. 80 min)*					
Marco	Male	50	Hoeksche Waard	Construction worker	No
Gerard	Male	66	Hoeksche Waard	Farmer	No
Edwin	Male	64	Hoeksche Waard	Livestock supplier	No
Roy	Male	66	Hoeksche Waard	Process operator	No
Jan	Male	81	Hoeksche Waard	Pensioner	No
Henricus	Male	61	Hoeksche Waard	Entrepreneur	No
Klaas	Male	72	Hoeksche Waard	Pensioner	No
*Interview 10 (location: living room; approx. 70 min)*					
Cornelis^c^	Male	74	Lansingerland	Teacher	No
Johanna	Female	69	Lansingerland	Pensioner	No
Benjamin	Male	80	Lansingerland	Pensioner	No
Helena	Female	79	Lansingerland	Pensioner	No
Piet	Male	69	Lansingerland	Pensioner	No
Carmen	Female	68	Lansingerland	Pensioner	No
*Interview 11 (location: living room; approx. 60 min)*					
Daan	Male	20	Hoeksche Waard	Logistics employee	No
Lotte	Female	19	Hoeksche Waard	Student^d^	No
*Interview 12 (location: living room; approx. 50 min)*					
Jermaine	Male	52	Rotterdam	Pipe fitter	Yes
Randall	Male	68	Rotterdam	Pensioner	Yes
*Interview 13 (location: living room; approx. 80 min)*					
Femke	Female	23	Westland	Student^d^	No
Romy	Female	23	Westland	Legal secretary	No
Max	Male	24	Westland	Grower	No
*Interview 14 (location: club building; approx. 60 min)*					
Michelle	Female	26	Westland	Student^d^	No
Kevin	Male	22	Westland	Student^d^	No
Bob	Male	22	Westland	Student^d^	No

^a^ We have generalized some occupations in order to safeguard the respondents’ anonymity.

^b^ Migration background is the official term used by Statistics Netherlands for a person of whom at least one parent was born abroad (Statistics Netherlands 2024b). All respondents with a migration background in interviews 1 and 7 arrived in the Netherlands only recently. The respondents with a migration background in interviews 3 and 12 have been living in the Netherlands for a longer period of time.

^c^ These respondents had obtained a degree at the university of applied sciences level (in Dutch: *hogeschool*).

^d^ Of all the respondents who were students at the time of the interview, only Kevin was enrolled at a university. Femke, Michelle, and Bob studied at a university of applied sciences (in Dutch: *hogeschool*). Lotte was still in high school.

Each interview followed a similar structure. First, we asked the respondents to take a moment to think about their initial associations with the concept of sustainability, which enabled us to identify which issues and interventions were most relevant to them. If not already discussed, and in addition to any other issues and interventions raised by the respondents themselves, we asked them to elaborate on their perspectives on green energy (e.g., wind turbines, fossil fuels); green environments (e.g., vegetation, gardens); and green lifestyles (e.g., recycling, meat consumption). These issues have been very dominant in public and political debate in the Netherlands, and most municipalities have developed interventions to tackle them. We asked each respondent to discuss who they thought would be the best fit for designing and executing interventions for a given issue. Thereafter, we invited the respondents to reflect on the sustainability interventions adopted in their municipality, and we discussed how this reflection linked to the prior perspectives that they shared. Importantly, all the respondents said they had enjoyed the (focus-group) interviews, and all of them were compensated with a €35 gift card at the end of the interview.

All interviews were conducted in Dutch. The interviews were recorded, transcribed, and pseudonymized and analyzed thematically using ATLAS.ti in an abductive, iterative process that enabled theoretical insights to be applied and explored during the analysis (Timmermans and Tavory 2012). After open coding, we connected recurring codes to form the overarching and comprehensive lenses detailed below, which we then located in extant literature to highlight their relevance. The quotes presented below were translated into English.

## Results

When asked to describe their initial associations with the concept of environmental sustainability, our respondents often mentioned, among other topics, electricity, solar panels, electric vehicles, high prices, nature, green environments, being economical with goods, pollution, or circularity. These associations reveal that many respondents did not predominantly link sustainability to global subjects like anthropogenic climate change, rising sea levels, or CO_2_ emissions. This reflects earlier suggestions that individuals without a tertiary education are typically less concerned about or aware of long-term global issues like climate change (see, e.g., Hoekstra et al. 2024; Kvaløy, Finseraas, and Listhaug 2012; Lee et al. 2015), even though these feature prominently in both the public debate and the sustainability interventions proposed by governments.

We identified three ways in which our respondents perceived sustainability issues and interventions, which we labeled *lenses*. The first and most prominent—*lens of need—*was used by our respondents when they turned their attention to how sustainability would impact their personal financial circumstances, health, or comfort. The second of the three lenses—*lens of community—*focuses on how sustainability aligns with the local community. This lens was especially pronounced when the respondents discussed the design and execution of interventions, expressing a strong preference for the involvement of community actors. The third lens—*lens of doubt—*highlights the doubts raised by sustainability issues and interventions and the actors proposing these interventions, which often led to disengagement and denying individual responsibility or relying on strategies to cope with their doubts.

### Lens of need: “where can I save?”

The most prominent lens was need, which was used whenever a respondent discussed how sustainability impacts their personal, everyday life. This lens links to one of the fundamental characteristics of the environment as “providing us with the resources necessary for meeting our material needs and wants, [serving] as our ‘supply depot’” as put forward by Dunlap (2010, 17). The perspectives uncovered among our respondents show how similar practical considerations shape how individuals relate sustainability to their personal needs.

Many respondents referred to financial needs, which, for instance, caused them to economize when it came to goods or energy. Most, and especially those respondents in lower-income positions, hoped that green energy interventions, in particular, would lower the cost of living, and they would thus be able to “profit from it,” as voiced by Jermaine. Engaging with sustainability was also believed to have health benefits, for instance, by reducing air pollution in cities caused by, for example, infrastructure and industries close to where the respondents resided. Likewise, Edwin called for “alternatives” to plastic because plastic “destroys us,” and Lia expressed her concern about her health by limiting her consumption of meat, which she regarded as “a burden to the body.” Other female respondents who, like Lia, had a migration background but arrived in the Netherlands only recently, similarly stood out by discussing food from the perspective of health, like Yasmin, who said that “eating a lot of red meat is not good for our body.” Another, albeit less urgent, need expressed by some respondents was comfort. Adding to research showing that fear of a loss of comfort inspires negative attitudes to sustainability (see, e.g., Jansma, Gosselt, and de Jong 2020; Stoll-Kleemann, O’Riordan, and Jaeger 2001), our work highlights that sustainability could also be perceived to *ensure* comfort. Green environments, in particular, were often said to be “pretty” (Firuza) or to offer opportunities for relaxation (Lotte) and a positive “ambiance” (Selam).

The above-illustrated needs for low costs, health, and comfort recur in prior literature on various isolated sustainability issues and interventions as well (see, e.g., Ebrahimigharehbaghi et al. 2019; Jansma, Gosselt, and de Jong 2020; Stanley, Wilson, and Milfont 2021), including studies on individuals of limited financial means (see, e.g., Laidley 2013). Our interviews highlight that these needs are expressed across sustainability issues and interventions. Tellingly, however, the respondents’ needs were seldom explicitly related to long-term goals like combating climate change. While some respondents acknowledged the importance of tackling matters like pollution or the loss of biodiversity, goals linked to addressing climate change were rarely perceived as an end in and of themselves. This resonates with prior indications that many people, and those who are less affluent in particular, are predominantly concerned about climate change for “proximal, personal or social reasons, such as convenience, saving money, or improving health” (Whitmarsh, O’Neill, and Lorenzoni 2013, 13; cf. Carfagna et al. 2014), which our interviews reveal applies across sustainability issues. Monica said that she does not often think about “the bigger picture” but is “always busy with saving…where can I save?” Likewise, in terms of the less urgent need for green living environments, Kevin’s view represented that of other respondents: he liked green environments not specifically because of the goal of biodiversity, but first and foremost because they are “prettier [and] cool the air.”

Following these needs, many of our respondents thought positively about sustainability issues and interventions when they met a personal everyday need (e.g., high living costs) and showed environmental concern when they were aware of environmental problems that affected them personally (e.g., pollution in their direct surroundings). The respondents typically wanted to contribute to interventions that they felt would meet their own needs, and some thought that increasing awareness of the urgency behind proposed sustainability interventions would mobilize others as well. Regarding solar panels, for instance, Max believed that “the government doesn’t have to do anything because it just saves money and people who have money will automatically invest in it.”

Yet, practical obstacles were mentioned to threaten this engagement, such as a lack of time and resources. Albert argued that “sustainability always equals higher tariffs,” which many of the respondents said they could not afford. These barriers led some to believe that sustainability, and participation in costly green energy interventions in particular, is exclusively for the rich. They “can pay for [solar panels], they get subsidy, but the workers can get the middle finger” (Ronald); or, as Jeffrey put it, “for normal people, it just doesn’t work.” These findings reflect the common observation made in earlier research that low-income individuals, many of whom have not completed a tertiary education, link sustainability to economic affluence. These studies suggest that being in a weaker economic position would reduce the degree of overall support for sustainability interventions and feed into the perspective that sustainability is “more of an upper-class thing” (Laidley 2013, 160; see, also, Lübke 2022). However, the lens of need, as our respondents employed it, highlights that having fewer resources does not necessarily have to inspire opposition to sustainability interventions or less concern for sustainability. If a sustainable alternative was cheaper, respondents expressed an interest in adopting it. Randall, for instance, said that he cooks on a gas stove but would “take [an induction stove] immediately” if it was cheaper. Similarly, our respondents’ discussion of the resources needed for many sustainability interventions was associated with calls to break down the financial barriers to participation, which a number of respondents sought to resolve by calling for subsidies that would enable people with lower incomes to also invest in or benefit from green energy.

Reflecting the role of practicalities and immediacy through the lens of need, many of our respondents demanded decisive action whenever they were convinced of the need to tackle a sustainability issue, but some believed the involved actors were often indecisive, and politicians in particular. This reflects a common critique about politicians among individuals without a tertiary education, who are known for both valuing straightforwardness and practical or hands-on interventions and for blaming the bureaucrats for their preoccupation with rules (Noordzij, de Koster, and van der Waal 2021; Visser, de Koster, and van der Waal 2023). These respondents also wanted interventions that “keep going” instead of those that just “muddle through” (Marja), citing those who say: “that’s how we’re going to do it and [it] won’t be [changed] after a couple of years” (Michelle). A similar critique on a lack of decisiveness was aimed at governments that asked too many actors for their input: “we got to a world where, when someone has a plan, about 7,000 people need to have a say about it” (Henricus).

Finally, the lens of need typically cuts both ways, and our respondents called to mind various instances that they thought justified having *less* concern for sustainability: burning fossil fuels to bring down energy prices (Klaas); using a car to take large items of trash for recycling (Lia); fossil-fueled machines that are needed for manual work, like a leaf blower (Selam) or sweeper (Marcel); or industries that produce crucial materials like steel (Robert). Jermaine, for instance, said that he did not find green environments that important because he experienced a pressing need to increase the number of parking spots: “trees are more annoying than helpful [because] you take spots where people could park.” Another recurring example came from the respondents who had blue-collar jobs involving strenuous physical labor. People who “work on the streets the entire day,” Jeffrey noted, “are happy to be home and heat up their house. You can’t expect these people to say: ‘well, you have to put your heater on 17 degrees [°C or 62 °F].’” Romy, who lived in a municipality with a large agricultural sector, also argued that eating meat is associated with blue-collar jobs because “gardeners work hard the entire day and afterward [want to eat] meat and mash.” Hence, while sustainability was often seen as relevant for achieving a practical need, at other times, it was perceived as an obstacle to fulfilling a different need.

Furthermore, indifference to sustainability issues and interventions seemed to arise in the *absence* of need. As Ingrid put it, sustainable behavior is a matter of “what bothers you and what doesn’t bother you.” Monica admitted that she “was never concerned with” sustainable energy until she had a financial need caused by the energy crisis that started in 2022 and was ongoing throughout the data-collection process. Nonetheless, a number of respondents were sympathetic toward interventions that would not require them to change their habits or did not really affect them in their everyday lives, such as using paper bags instead of plastic ones. This highlights the role played by habits or routine behavior in how sustainability interventions are embraced (see, e.g., Whitmarsh, O’Neill, and Lorenzoni 2013), especially when the need for their introduction is perceived to be low.

### Lens of community: “improving the livability-sustainability”

While the lens of need primarily concerned sustainability in the context of the personal lives of the respondents, the second lens shifted the focus to their communities. This lens possibly plays a further part in our earlier observation that the respondents were less engaged with global issues like climate change and that they likely regarded them as distant problems that do not affect their local community. However, this lens highlights the relevance of a physical or spatial dimension in the emergence of environmental challenges (Dunlap 2010) as well as in individuals’ engagement with these challenges or support for interventions (see, e.g., Clarke et al. 2016; Liebe and Dobers 2019), and touches on a strong sense of collective identity and action which frames how sustainability issues and interventions are perceived (see, e.g., Amann and Doidge 2023; Diamond 2023; Goossens, Oosterlynck, and Bradt 2020).

Very much like the lens of need, that of community was employed to determine the perceived benefits of sustainability. However, unlike the lens of need, this one was used to consider how sustainability benefits the community they cared about (e.g., their town, neighborhood, or club). Jermaine’s opinion on wind turbines reflected that of many other respondents, who were positive about their possible construction close to where they lived, if it meant cheaper electricity for those residing there. In these circumstances, “it would provide something for the neighborhood and can improve the livability-sustainability.” Jermaine, who has a migration background, further showcased his perception of “livability-sustainability” by criticizing the absence of a communal approach to practical needs and comfort in the Netherlands compared to the country where he was born, reflecting migrants’ unique position to connect experiences across different contexts.

Jermaine: [But] I was thinking about how they should build houses in such a way that it is not too expensive to build them, so that people with lower incomes can live in a decent house because in the Netherlands—I’m from [anonymized place of birth] and there you have social housing, you have a neighborhood with real social houses, there. I think that those social houses are better for [people]…But then you arrive in the Netherlands and they say social houses—where are the social houses? They say that the old houses [are] social houses but I say that is not the social house I’m used to.

Interviewer: But what is your idea of social housing?

Jermaine: Let’s say, a real apartment building that—isn’t that expensive. Everybody knows that it is cold in the Netherlands at the moment—they have central heating…And it would be nice to build an apartment building with those people, for those people, and then build an office so that we can help them get a job…

Interviewer: So, all in once?

Jermaine: All in once…People [should] live in a good house—to give an example, a trash man has a tough job [and] then he comes home and that house is all cold—but if he would come home, and it is pleasantly warm…he thinks: “I am looking forward to working tomorrow.”

Respondents typically applauded sustainability interventions whose design and implementation were sensitive to the sociocultural makeup, outlook, and preferences of their communities. Simone explained, for instance, that “generating energy for the neighborhood ensures support [because] it makes it more personal…when it becomes too big, you lose the connection.” One example of a sensitive approach concerned the location of wind turbines in the municipality of Hoeksche Waard, which is located on an island where residents generally have a strong sense of (rural) community. Because these wind turbines were located “on the outskirts [of the island]” and kept the mainland free from wind turbines, they were “not in anyone’s way” (Daan), and none of the respondents from this area strongly opposed their construction.

The lens of community also extended to perspectives on actors, and respondents generally preferred involving actors who came from their communities. The best fit for designing and executing sustainability interventions would be role models (Monica), “one person who represents your town” (Romy), “town’s people who the entire town likes” (Femke), “the people on the work floor” (Marja), or “people who started at the bottom” (Ronald). This is because they would “focus on us” (Romy), be sensitive to the problems people face (Jermaine), or “have a heart for the city” (Ronald). Consequently, the municipal level of government was also repeatedly described as being the go-to governmental body for proposing and executing a significant number of sustainability interventions since they “stand closer to the people” (Antoinette), despite the criticisms this level of government also received, as detailed below.

The perspectives above help to understand why some respondents criticized interventions that they perceived to be *insensitive* to the community. Mindful of the preferences of the people in their municipality, Monica and Femke raised doubts about the effectiveness of sustainability interventions that did not reflect the “mentality of the neighborhood” (Monica) or failed to consider that most people believe that many sustainability issues are “all bullshit” (Femke). Discussing the waste-collection system in their municipality, which had received considerable local opposition, Femke and Romy wondered “whether they installed it in consultation with a citizen council, for instance” (Femke). If the community had been involved in the decision-making process, the municipality would have gotten “a view of what we find important [and] not only from their own…higher position” (Romy). This sentiment reflects that identified by Diamond (2023, 511; italics in original) in her rural respondents, who believed that environmental policies “are too often done *to* them and not *with* or *for* them.”

Actors who would fail to consider the local community were also seen as insensitive. A number of respondents voiced such criticisms about politicians in particular because they were perceived to “have little practical working experience” (Edwin). Max, who is a farmer in the municipality of Westland, linked his criticism of sustainability interventions for agriculture to a perceived distance of policymakers who “don’t see the problems of their [decisions], because they are not active in the sector. There are companies that are in the sector and have much more expertise.” Others directed their critique to other actors, like big businesses or energy corporations: “they are too big, and then they don’t listen” (Irma). Compared to “the municipality [that] does not need to make a profit,” energy companies make “outrageous profits on the backs of us all” (Dirk). Despite their overall criticism of the insensitivity of a wide range of actors, some of our respondents did allocate a role to them in interventions as long as the local community has a seat at the table. Discussing citizens’ initiatives, Cornelis explained that “you need to include normal people, and it is okay to add scientists [and] the CEO of a bank—but it needs to be a mixed group that is not bound to big economic interests, the big power interests.”

### Lens of doubt: “saving one thing but depleting something else”

The issue of sustainability raised several doubts among our respondents. These were most prevalent in discussions on the complexity inherent to many sustainability issues and interventions (see, also, Rhein and Bernauer 2025). Their misgivings often addressed the perceived technical and practical limitations or downsides of sustainability issues and interventions—for example, electric vehicles are perceived to be dangerously silent, waste-collection systems are regarded as impractical, and plant-based diets are believed to be unhealthy—as well as whether sustainability interventions are, ultimately, genuinely sustainable at all. For instance, respondents asked about whether the batteries of electric vehicles could be recycled, what happens with the raw materials of wind turbines after they cease to function, and what the ecological footprint of foods popular in plant-based diets is (e.g., avocados).

The type of information further exacerbated doubts many respondents said they had, which was said to be, for instance, incomplete or in conflict with other facts they came across. Yasamin found it hard to believe that meat consumption had a negative impact on the climate: “why would that be a bad thing? I don’t have enough information [to judge that].” These misgivings regarding the information respondents said to receive, or lack thereof, were expressed both by respondents who showed difficulty in obtaining or comprehending the information and by those who showed great awareness of the complexity and multifaceted nature of sustainability. The latter is illustrated by Monica and Lia, who doubted whether green energy and electric vehicles are actually sustainable following the conflicting and incomplete information they received about these complex issues, inciting great skepticism.

Lia: I recently heard that those electric vehicles are not so sustainable…and that they are used as the solution, but then I hear that it is not the solution. [Monica started laughing]

Interviewer: Monica, you had to laugh—is this also something that you hear?

Monica: Yes! Yes, and building a battery costs such an immense amount of resources…That is anything but environmentally conscious. Yet we don’t have a choice anymore, we cannot keep using natural gas and gasoline and crude oil. It is only reasonable that we go electric, but there is a façade regarding how environmentally conscious that electric vehicle is…

Interviewer: But what does that do to you when you see that it’s apparently not as good?

Monica: May I say that honestly?…I feel like we’re being fooled a bit sometimes…And I am not sure—because I once read how much resources we need for one battery, so then we are trying to save one thing, but on the other hand, we are depleting something else. And there is no discussion about how much that battery costs, only about how bad gasoline is and how bad oil is and how bad CO_2_ emissions are and how bad that all is—but that battery!

Respondents also frequently raised doubts about the actors proposing and executing sustainability interventions, which seems to stem from a general deep-seated mistrust of those actors and highlights that support for interventions depends on trust in the actors proposing them (see also Jansma, Gosselt, and de Jong 2020). Beyond the perceived indecisiveness and insensitivity of many actors, as noted in the previous sections, many respondents also doubted these actors, and politicians in particular, as well as their actions. The interview with a group of men in a garden shed in the municipality of Hoeksche Waard showcased how a general mistrust of the interests of politicians spilled over into skepticism regarding sustainability interventions. Through the lens of community, Edwin supported specific local initiatives that were sensitive to the demands of the community, yet he doubted whether this was possible most of the time.

Edwin: But about that wind turbine located in the Philipsdam, when you drive to [the province of] Zeeland. People from the surrounding area have been involved and profit from it financially, and they laid down the demand that the turbine stops when a bird of prey comes close…

Interviewer: If I understand Edwin correctly, you say that when residents in the area lay down such a demand, that the municipality should listen to it and that it should just happen…

Edwin: Yes…But that doesn’t happen nine out of ten times.

Interviewer: And why is that, do you think?

Edwin: Well, then again there are so many different interests in politics—those guys who put [the turbines] there have so many interests and they are in a position of power and just do it. And maybe, and I shouldn’t say this, but politics [involves] a conflict of interests.

Piet voiced what was another doubt about the actions of actors, namely the perceived tendency of politicians in particular to not deliver on their promises: they “too easily say we are going to do it all, we’re going to do this, we’re going to do that—while they can’t achieve it.” Furthermore, Marcel showed how actors were also believed to be inconsistent: his municipality first “cuts down a kilometer of forest for a highway, and then [starts] complaining that there should be more trees—give me a break!” Similar frustration was expressed regarding the recurring perception that politicians failed to maintain their own sustainability standards, especially when they were perceived to “burden us with all kinds of rules” (Henricus). “If you want to go on a holiday for a week, you have to go broke,” Marcel said. Politicians, however, “go up in the air 20 times a year…they are allowed to fly, we aren’t—it makes my blood boil!” Crucially, many of the doubts above do not simply involve a knowledge deficit, as is commonly alleged (cf. Hoekstra et al. 2024; Hornsey et al. 2016), but also spring from frustration with the actual outputs and outcomes of the actors involved.

Furthermore, as reflected in Marcel’s comment above, doubts raised against sustainability issues and solutions, and the actors proposing them, were also fed by the feeling that some sustainability interventions are meddlesome and imposed on people. In these instances, sustainability interventions sparked resentment because they were interpreted as “invasive and obnoxious critiques of personal behavior” (Laidley 2013, 160–161; see, also, Van Meurs et al. 2022). Piet, for instance, said that “the municipality, government, the authorities, government levels—they should offer possibilities [to act sustainably], they should not be moralistic like ‘this is how it must be done.’” Femke, when discussing green lifestyles she personally engages with less, similarly expressed her appreciation for the adage “live and let live.” The resistance against interventions that are interpreted as meddlesome was also clearly showcased by the group of women who discussed meat consumption and contrasted top-down repertoires with inclusive ones that would not breed resentment.

Paulina: I eat a piece of meat every day. Not a chunk…but just a meatball…

Interviewer: But what do you think when you hear a politician say that you should stop eating meat?

Anna: Well, terrible…

Paulina: I think that’s terrible. Well, and then my food doesn’t taste good…

Interviewer: But why is it terrible?

Anna: Well, that they impose on you how much or how little you are allowed to…

Paulina: They just want to impose on you what you must do. What you are allowed to eat. Where you are allowed to go to…It’s forced. Like we must all stop eating meat, we must all become vegetarian.

Aaltje: Yes. But…*c’est le ton qui fait la musique* [it’s the tone that makes the music]. You could also say: Wouldn’t it be better to for your health to eat only a small piece…

Our respondents principally dealt with their doubts in two ways. First, some respondents suggested that they themselves bore little responsibility for the issues discussed. Resonating with earlier studies that have highlighted a tendency to deny having any personal responsibility for climate-change interventions, instead “point[ing] the finger at larger systems and powerful actors” (Kennedy and Givens 2019, 660; see, also, Stanley, Wilson, and Milfont 2021; Stoll-Kleemann, O’Riordan, and Jaeger 2001), our interviews demonstrate that this type of response is a typical expression of doubt and that it is linked to a wide range of sustainability issues and interventions. These respondents pointed toward actors or groups with more responsibility than they have, such as youngsters who do not do their fair share, countries that pollute much more than the Netherlands, businesspeople who take short flights, or companies that have a large ecological footprint. “Who is polluting the Netherlands?” Randall asked, “don’t say the cars—it is Shell…the industry pollutes more than cars.” Daan, likewise, said “me [and] my family can contribute, but in the bigger picture we can’t really [contribute].”

Second, respondents often relied on strategies to cope with their doubts (cf. Rhein and Bernauer 2025; Stanley, Wilson, and Milfont 2021; Stoll-Kleemann, O’Riordan, and Jaeger 2001). One way of doing this is linked to some of our respondents’ above-mentioned critiques on issues and interventions that they felt were imposed on them. Respondents stressed the impact of small contributions while also opposing radical (e.g., closing an airport, plant-based diets), fast-paced interventions, or issues that would receive a disproportionate amount of attention—or “overkill” (Piet). Femke, for instance, said that “when [sustainability] goes step by step, then I think it is okay but [not] when it comes, abruptly, radically.” Gerard, likewise, stressed that large solar fields are not required when “people can do more themselves using solar panels—on roofs, no matter how small.” Another strategy linked to the complexity and downsides of sustainability was calling for innovation. We observed this tendency, especially among male respondents. Frank, for instance, asked for the development of silent planes to reduce the noise pollution of airports, while Bernard addressed new fishing technologies that would improve the industry’s sustainability. A final way in which some respondents coped with doubts was by calling for practical, clear-cut, and transparent information provision by key actors (e.g., scientists, experts with know-how, governments) that would aid them in navigating or alleviating these doubts, which extends the established role of high-quality factual information in garnering support for and participation in sustainability interventions reflected in the information-based approaches (cf. Ebrahimigharehbaghi et al. 2019; Hinton and Goodman 2010; Hornsey et al. 2016; Kvaløy, Finseraas, and Listhaug 2012; Pisello et al. 2017).

## Discussion and conclusion

Using in-depth focus-group interviews (*n* = 57), we aimed to add to the extant environmental-sociological literature by examining the perspectives individuals have of environmental sustainability through their own eyes while also accounting for the multifaceted nature of their positions across a wide range of sustainability issues and interventions. Our study mainly focused on the perspectives of non-tertiary educated individuals, and we identified three popular perspectives.

The first and most prominent lens—*lens of need—*was employed by the respondents to express their need for sustainability issues and interventions that improved their financial circumstances, health, or comfort. While respondents who used this lens demonstrated personal engagement with sustainability, many identified (financial) barriers to their participation. Conversely, in the absence of need, the respondents could be indifferent toward sustainability. Additionally, respondents mentioned circumstances where fewer considerations of sustainability were required. The second lens—*lens of community—*was employed to assess how sustainability aligns with their local community. This revealed a strong preference for the involvement of community actors in proposing and executing interventions, including the municipality. The third lens—*lens of doubt—*was associated with the technical and practical doubts about numerous sustainability issues and interventions, as well as the information provided to them. They likewise expressed misgivings concerning the perceived intentions behind the consistency and sincerity of the actions of actors and those of politicians in particular. These doubts led many respondents to disengage with sustainability or deny individual responsibility, while others resorted to coping strategies like calling for innovation.

It is worth noting that these three lenses largely align with attitudes and motivations identified in previous literature on sustainability issues, as highlighted at various points in our analysis. Nonetheless, researchers have often studied these issues in isolation and have not pursued their investigations from the perspectives of individuals themselves. Our approach—studying individuals’ perspectives through their own eyes while accounting for the multifaceted nature of these perspectives across a wide range of sustainability issues and interventions—has at least four implications while simultaneously inspiring novel avenues for future research.

First, we found that global issues like combating anthropogenic climate change or cutting CO_2_ emissions were not the priority for many of our respondents. Conversely, we found that many respondents were principally engaged with sustainability issues and interventions that were of immediate relevance to and had practical implications for their personal lives or community. This necessitates a research agenda with a broader analytical scope that recenters research into issues beyond climate change and explores how individuals’ wider sets of sustainability attitudes and behavior are linked to their views on climate change. Part of this agenda requires us to explore among the population at large whether and why the lenses we have identified are, despite their applicability across issues and interventions, more strongly linked to some issues or interventions than to others, which are beyond the scope of our current study.

Second, earlier research, especially that tied to prior work on environmental inequality (see, for an overview, Dunlap 2010), commonly links the cultural lifestyles (see, e.g., Geerts, Vandermoere, and Oosterlynck 2023) and weaker economic positions (see, e.g., Lübke 2022) of individuals without a tertiary education to their limited concern for and engagement with sustainability. Our analysis, however, highlights that these individuals’ perspectives are hard to label uniformly as either opposition or disengagement. Although many respondents faced financial barriers to engaging with sustainability, criticized politicians whom they perceived to be insensitive to their community, or denied individual responsibility in the face of doubt, we revealed varying levels of concern and engagement depending on the issue or intervention under discussion and the lens through which they were viewed. Even those respondents who might typically be described as “eco-powerless” (Kennedy and Givens 2019) in the face of their doubts, engaged positively with some issues and interventions when they had a direct need for them or they thought would benefit their community.

Third, our sociological approach puts individuals’ perspectives on a wide array of sustainability issues and interventions center stage and contextualizes them within both their material surroundings and social and cultural life-worlds. This approach proved its relevance in uncovering the various ways in which our respondents perceived sustainability, highlighting that their perspectives were not fixed. The toolkit approach (Swidler 1986) has particular relevance for further exploration of this observation because it was developed to illustrate how individuals navigate diverse situations by shifting between a wider set of distinct, readily available narratives, perspectives, and meanings—or “strategies of action” (273). Similarly, our respondents typically used all three lenses, and all three lenses were adopted to view the same issue or intervention from different angles. There is a need to systematically explore further how, why, and in what institutional, cultural, or relational contexts different lenses are employed. In addition, how individuals navigate and consolidate different or conflicting perspectives is an empirical question with relevance for future investigations.

Finally, future research could explore the social bases of the three lenses by uncovering both the unique role different social characteristics play in shaping individuals’ perspectives and their relevance among the population at large. While it is beyond the scope of our qualitative study to systematically identify and interpret the role of social characteristics, it stands to reason that different social groups employ the uncovered lenses to varying extents. So, those facing heightened economic insecurity might predominantly appeal to the lens of need when discussing the implementation of green energy, just as individuals who prioritize personal health may be more inclined to use this lens when discussing sustainable diets. Furthermore, having a strong rural or regional identity or a robust sense of community could be linked to the use of the lens of community. Other approaches would stipulate that those with more scientific or sustainability-specific knowledge express less doubt, similar to those whose cultural and material resources enable them to better navigate sustainability amid complexity and uncertainty.

Regarding the future scrutiny of the social bases of the three lenses, the role of migration background stood out in our study. Our analysis suggests that some perspectives are uniquely tied to the experiences of respondents with a migration background. The female respondents with a migration background, for instance, each discussed sustainable eating habits from the perspective of health, while another respondent with a migration background presented a communal approach to sustainability that he tied to his country of birth. This adds to prior research highlighting, for instance, the important role of (traditional) food in new social settings (Visser, Bailey, and Meijering 2015) and the stronger preference among migrants for high-quality and healthy (traditional) food (Berggreen-Clausen et al. 2022). In short, our findings clearly warrant future research on the social bases of the three lenses and how they relate to various sustainability issues and interventions in general, as well as the role of migration background specifically.

Our study provides valuable insights for professionals and governments grappling with the challenges of garnering public support for sustainability interventions. Just like the work of Hinton and Goodman (2010, 250), who focused on sustainable consumption, our findings justify criticizing the dominant information-based approaches to changing attitudes and behavior for “[ignoring] the often unequal structural, institutional and cultural frameworks within which we make our consumption decisions.” Specifically, our sociological approach to sustainability perspectives emphasizes pathways to expanding support for sustainability interventions by increasing their sensitivity to the uncovered lenses of need, community, and doubt. This includes: 1) identifying and connecting to individuals’ immediate and practical needs in their personal lives; 2) integrating interventions within individuals’ communities; and 3) ensuring the transparency and consistency of goals, information, and actions.

Our findings also highlight that decision-making requires sensitivity to the sustainability issue or intervention at hand and the lens through which it is viewed, which helps to decide which actors to involve (e.g., local actors or experts), the pace of the intervention (e.g., taking decisive action without much prior participation or step-by-step interventions that consult multiple individuals), the issue to be tackled (e.g., immediate (local) or global ones), or barriers to participation that need to be dismantled (e.g., material barriers or complex facts). Rarely, thus, does our study show that individuals support sustainability interventions unconditionally. Yet, conditional support might be strong when interventions match the perspectives individuals have of sustainability.

Ultimately, these insights could help to rethink sustainability interventions to make them more inclusive of popular perspectives, and those held by non-tertiary educated individuals in particular. Our findings include the demonstration that global goals linked to combating climate change may not resonate with this majority sociodemographic, but they might think more favorably about interventions associated with immediate, personal sustainability that contribute nonetheless to meeting these goals. In addition, while a governance perspective makes it crucial to involve regional governmental actors to connect different stakeholders in sustainability projects, the lens of community used by our respondents highlights that they must convince individuals of their sensitivity to their community or, preferably, that they are part of it. Moreover, the dominant information-based approaches that seek to ensure support for sustainability are unlikely to succeed when the information presented feeds into existing doubts or creates new ones, or when it is provided by actors who are deemed to be inexperienced or have limited expertise.

An approach that is sensitive to popular perspectives also provides pathways or strategies to handle support for sustainability issues and interventions that many individuals resist or oppose. We found that opposition often stemmed from a lack of perceived need, remote initiatives and actors, or ongoing doubts. Policymakers could ensure that interventions that individuals do not feel they need do not exert a negative impact on their everyday lives. Existing uncertainties could also be alleviated by emphasizing the innovative nature of the proposed interventions or by presenting transparent information when facts appear to be in conflict. Likewise, nature-based interventions that appeal to the many individuals’ liking of green environments or replacing disposables with reusables in a manner that does not require a change of habits are likely to have wide approval, even though individuals do not experience a direct need to tackle the related issues like climate change.

## Notes

1 We asked the interviewees to invite acquaintances or family members to also attend their interview. As a result, one or more respondents in some groups had completed a tertiary education at the university of applied sciences level (in Dutch: *hogeschool*).

2 Our study is co-funded by six municipalities in the Netherlands, which is where our interviews were conducted. These municipalities are all located in the densely populated, economic center of the country (*Randstad*) and include both urban municipalities, i.e., Den Haag (approx. 550,000 inhabitants) and Rotterdam (approx. 660,000 inhabitants), as well as rural ones, i.e., Hoeksche Waard (16 towns; approx. 90,000 inhabitants in total), Lansingerland (three towns; approx. 60,000 inhabitants in total), Voorne aan Zee (16 towns; approx. 70,000 inhabitants in total), and Westland (11 towns; approx. 110,000 inhabitants in total). Despite being close geographically, these municipalities differ demographically, economically, and culturally.
